# Topology-based metrics for finding the optimal sparsity in gene regulatory network inference

**DOI:** 10.1093/bioinformatics/btaf120

**Published:** 2025-03-24

**Authors:** Nils Lundqvist, Mateusz Garbulowski, Thomas Hillerton, Erik L L Sonnhammer

**Affiliations:** Department of Biochemistry and Biophysics, Stockholm University, Science for Life Laboratory, Solna 171 21, Sweden; Department of Biochemistry and Biophysics, Stockholm University, Science for Life Laboratory, Solna 171 21, Sweden; Department of Biochemistry and Biophysics, Stockholm University, Science for Life Laboratory, Solna 171 21, Sweden; Department of Biochemistry and Biophysics, Stockholm University, Science for Life Laboratory, Solna 171 21, Sweden

## Abstract

**Motivation:**

Gene regulatory network (GRN) inference is a complex task aiming to unravel regulatory interactions between genes in a cell. A major shortcoming of most GRN inference methods is that they do not attempt to find the optimal sparsity, i.e. the single best GRN, which is important when applying GRN inference in a real situation. Instead, the sparsity tends to be controlled by an arbitrarily set hyperparameter.

**Results:**

In this paper, two new methods for predicting the optimal sparsity of GRNs are formulated and benchmarked on simulated perturbation-based gene expression data using four GRN inference methods: LASSO, Zscore, LSCON, and GENIE3. Both sparsity prediction methods are defined using the hypothesis that the topology of real GRNs is scale-free, and are evaluated based on their ability to predict the sparsity of the true GRN. The results show that the new topology-based approaches reliably predict a sparsity close to the true one. This ability is valuable for real-world applications where a single GRN is inferred from real data. In such situations, it is vital to be able to infer a GRN with the correct sparsity.

**Availability and implementation:**

https://bitbucket.org/sonnhammergrni/powerlaw_sparsity/ and https://codeocean.com/capsule/4393635/.

## 1 Introduction

Gene regulatory network (GRN) inference has found many applications in systems biology, for instance, to characterize mechanisms in diseases such as cancer (Lopes-Ramos *et al.* 2018, Seçilmiş *et al.* 2020) or COVID-19 (Guzzi *et al.* 2020). Nevertheless, methods used for GRN inference (GRNI) vary in mathematical and biological model assumptions as well as the usage of the experiment design as input data (Seçilmiş *et al.* 2022a). What these methods tend to have in common, however, is the use of a hyperparameter to control the sparsity, i.e. the average node degree in the GRN. In a real biological application, it is important to estimate the single most correct GRN, which can be used to visualize and analyze the regulatory mechanisms and serve as a guide for further experiments. It has been shown that networks in living systems are typically sparse, leading to explorability and stability (Busiello *et al.* 2017). Hence, it is crucial to estimate a proper sparsity while inferring a GRN. This problem has been recognized and approached as part of the inference step with GRNI methods such as C3NET (Altay and Emmert-Streib 2010) where sparse GRNs are inferred by choosing interactions with significant mutual information. However, in practice the level of significance still serves as a hyperparameter and the problem of accurately optimizing the sparsity remains.

Choosing the hyperparameter value that best recreates the true GRN is a challenge due to a number of factors, such as not knowing the true sparsity of the real GRN, not being able to consistently measure prediction accuracy, e.g. via cross-validation, and the general lack of knowledge about the true interactions. Examples of previous methods for selecting optimal sparsity include the SPA workflow (Seçilmiş *et al.* 2022b) where a GRN information criterion (GRNIC) was formulated and tested on small GRNs with low sparsity, and an approach based on leave one out cross optimization (Tjärnberg *et al.* 2013). Both of these approaches suffer from instability issues, however, especially when the noise levels are high.

Although there are many unknown factors connected to any given GRN, a general assumption regarding the topology of GRNs is that they are scale-free (Zhu *et al.* 2007, Ouma *et al.* 2018). This has been shown to hold for real GRNs from several different sources (Garbulowski *et al.* 2024). The scale-free topology can be described by an approximate discrete power law distribution where the node out-degrees (number of regulatory interactions) are compared against their frequencies, and such a topology frequently appears in networks generated by nature (Rak and Rak 2020). We therefore decided to exploit the assumption of scale-freeness as the foundation for two sparsity selection methodologies to select the best GRN in terms of topology.

The goal of this study was to develop algorithms that increase the selection accuracy of GRN inference based on network topology. We propose two approaches: “goodness of fit” and “logarithmic linearity.” They both use adherence of the node out-degree distribution to a power law distribution as the main principle but use different statistical models. As the power law distribution should not involve the in-degree of nodes in GRNs (Almaas and Barabási 2006), only the out-degree was considered. We benchmarked these algorithms against gold standard GRNs for varying noise levels, sparsities, and inference methods. The results of the benchmark indicate that both algorithms are capable of selecting the GRN with a close to correct sparsity.

Most GRN inference algorithms do not address the issue of sparsity but instead infer a fully connected GRN. This GRN can be made sparser by setting all links with a score below a cutoff value to zero, but there is no guideline for how to choose the cutoff. It is to this end that we developed the two new algorithms presented here. In a real-world application, such as the inference of a GRN from cancer data, it is vital to be able to infer a GRN with the correct sparsity to represent the biological GRN most faithfully and to avoid false negatives and positives as much as possible. As GRN analysis of biological processes is becoming an increasingly popular tool in the omics era, this capability will be highly useful when using real data with the goal to identify new mechanisms that cause biological phenomena such as cancerogenesis. At the same time, the presented algorithms are only selecting GRNs from a range of varying sparsities, hence the accuracy of the selected GRN can only be as good as the applied inference method. With the continued development of better GRN inference methods, the new sparsity selection methods will improve our ability to correctly infer GRNs from real data, which will lead to new insights of the underlying regulatory mechanisms and ultimately to better therapies.

## 2 Materials and methods

The discrete power law distribution approximately describing the scale-free topology is defined by the following probability function $p_{X}(k)$ (Barabási 2009):

(1) $$p_{X}(k)=\frac{1}{\zeta (\alpha ,k_{\min})}k^{-\alpha },k\geq k_{\min}$$

where the inverse of the normalizing constant

(2) $$\zeta (\alpha ,k_{\min})=\sum_{n=0}^{\infty }{\left(n+k_{\min}\right)}^{-\alpha }$$

is the Hurwitz Zeta function with parameters α and $k_{\min}$. In this study, k represents the out-degree of a node (gene) in a GRN and $k_{\min}$ is set to 1, assuming a discrete power law distribution for all positive node out-degrees.

One interesting property of the discrete power law distribution follows from taking the logarithm of the probability function defined in Equation (1)
 

(3) $$\log(p_{X}(k))=\log(\frac{1}{\zeta (\alpha ,k_{\min})}k^{-\alpha })=-\alpha \log(k)-\log(\zeta (\alpha ,k_{\min}))$$

This proportional relationship reflects what is often considered to be a scale-free topology—there is a negative linear trend between the logarithm of the observed frequencies of nodes with a given number of links and the logarithm of the number of links per node.

Two methods are formulated below to test the potential of the hypothesis of true GRNs being scale-free in sparsity selection. They are both based on the same hypothesis but use different approaches.

### 2.1 The goodness of fit metric

The goal of GRNI is to infer a GRN as close as possible to the true one. A GRN is represented by the interaction matrix $A\in R^{n\times n}$ where n is the number of genes in the GRN and the element in the ith row and jth column of A represents the regulatory effect of gene i on gene j. Given a GRNI method with a set of hyperparameter values $\lambda_{g}\in \{\lambda_{1},\ldots ,\lambda_{G}\}$ that control the sparsity of the prediction, ${\hat{A}}_{g}$ is the inferred GRN generated with hyperparameter value $\lambda_{g}$. For such a GRN, the observed out-degree distribution can be obtained by counting the number of nonzero elements per row of ${\hat{A}}_{g}$
 

(4) $$c_{i}^{\left(g\right)}=\sum_{j=1}^{n}\;|s\mathrm{ign}(a_{i,j}^{\left(g\right)})|$$

where $c_{i}^{\left(g\right)}$ is the out-degree of gene i, n is the number of genes in the system and $a_{i,j}^{\left(g\right)}$ is the element in row i and column j of ${\hat{A}}_{g}$. Next, count the frequency of out-degrees $x_{d}^{\left(g\right)}$ according to

(5) $$x_{d}^{\left(g\right)}=\sum_{i=1}^{n}1_{d}(c_{i}^{\left(g\right)})$$

where 1≤d≤n is the range of out-degrees. Basic statistical theory (Jackson Hall and Oakes 2023) says that if the node out-degree of the GRN represented by ${\hat{A}}_{g}$ follows a discrete power law distribution, the goodness of fit statistic

(6) $$Q_{g}=\sum_{d=1}^{n}\frac{{\left(x_{d}^{\left(g\right)}-n^{\left(g\right)}p_{X}^{\left(g\right)}(d)\right)}^{2}}{n^{\left(g\right)}p_{X}^{\left(g\right)}(d)}$$

will be approximately $\chi^{2}$ distributed, where $n^{\left(g\right)}$ is the number of genes in ${\hat{A}}_{g}$ with a positive observed out-degree:

(7) $$n^{\left(g\right)}=\sum_{i=1}^{n}\;|s\mathrm{ign}(c_{i}^{\left(g\right)})|$$

and

(8) $$p_{X}^{\left(g\right)}(d)=\frac{1}{\zeta (\alpha_{ML}^{\left(g\right)})}d^{-\alpha_{ML}^{\left(g\right)}}$$

where $\alpha_{ML}^{\left(g\right)}$ is an approximation of the Maximum Likelihood estimator of α for GRN${\hat{A}}_{g}$, given by (Clauset *et al.* 2009):

(9) $$\alpha_{ML}^{\left(g\right)}=1+n{\left[\sum_{i=1}^{n}ln(\frac{c_{i}^{\left(g\right)}}{k_{\min}-\frac{1}{2}})\right]}^{-1}$$

Even though the GRN topology is not assumed to exactly follow a discrete power law distribution, it can be assumed to do so asymptotically. The goodness of fit metric $Q_{g}$ will therefore take on small values for GRNs with link degrees close to such a distribution. The idea therefore is to investigate if the equation:

(10) $$\hat{A}=\mathrm{argmi}n_{{\hat{A}}_{g}}Q_{g}$$

holds any merit for predicting sparsity, i.e. if $\hat{A}$ is the inferred GRN closest in sparsity to the true one.

### 2.2 Logarithmic linearity metric

The logarithmic property shown in Equation (3) can also be used as a measure for the topology of the inferred GRN. Instead of the goodness of fit approach, one can look at how the logarithm of the observed frequencies of out-degree in the GRN relates to the logarithm of the out-degree. If the observed distribution is close to a power law, there should be a negative linear trend between the two. To measure the linearity, Pearson's correlation coefficient r(x,y) is used. This is defined for two stochastic variables X and Y as

(11) $$r(x,y)=\frac{\sum_{i=1}^{n}\left(x_{i}-\bar{x}\right)(y_{i}-\bar{y})}{\sqrt{\sum_{i=1}^{n}{\left(x_{i}-\bar{x}\right)}^{2}}\sqrt{\sum_{i=1}^{n}{\left(y_{i}-\bar{y}\right)}^{2}}}$$

With the notation used in the goodness of fit method, the correlation coefficient of interest becomes

(12) $$r(\alpha^{\left(g\right)},\beta^{\left(g\right)})=\frac{\sum_{d\in D^{\left(g\right)}}\left(\alpha_{d}^{\left(g\right)}-{\bar{\alpha }}^{\left(g\right)})(\beta_{d}^{\left(g\right)}-{\bar{\beta }}^{\left(g\right)}\right)}{\sqrt{\sum_{d\in D^{\left(g\right)}}{\left(\alpha_{d}^{\left(g\right)}-{\bar{\alpha }}^{\left(g\right)}\right)}^{2}}\sqrt{\sum_{d\in D^{\left(g\right)}}{\left(\beta_{d}^{\left(g\right)}-{\bar{\beta }}^{\left(g\right)}\right)}^{2}}}$$

where $\alpha_{d}^{\left(g\right)}=\log(x_{d}^{\left(g\right)})$, $\beta_{d}^{\left(g\right)}=\log(d)$ and $D^{\left(g\right)}=\{d:x_{d}^{\left(g\right)}>0\}$.

Since a close to scale-free GRN should show a negative trend in the logarithmic setting, a coefficient value $r(\alpha^{\left(g\right)},\beta^{\left(g\right)})=-1$ would be optimal. However, there is a risk that the correlation metric is close to −1 even for inferred GRNs with a lower sparsity than the true one due to the decreasing number of data points in $D^{\left(g\right)}$. To tackle this issue the *P*-value, $p_{g}$, of the correlation metric $r(\alpha^{\left(g\right)},\beta^{\left(g\right)})$ is used. Here the *P*-value is the probability of the null hypothesis that there is no linear relationship between the data series. To filter out GRNs that generate a strong positive correlation only cases where the correlation is less than −0.5 are considered. The objective becomes to investigate if the equation

(13) $$\hat{A}=\mathrm{argmi}n_{{\hat{A}}_{g}}p_{g},\;\text{so}\;\text{that}\;\;r(\alpha^{\left(g\right)},\beta^{\left(g\right)})<-0.5$$

holds any merit for predicting the sparsity. This is done in the same fashion as with the goodness of fit approach.

### 2.3 Workflow

To test the robustness of the new methods, we benchmarked them on GRNs inferred by four different GRNI methods: Least Absolute Shrinkage and Selection Operator (LASSO), Zscore, Least Squares Cut-Off with Normalization (LSCON), and GEne Network Inference with Ensemble of trees 3 (GENIE3), which are included in the GeneSPIDER package (Tjärnberg *et al.* 2017). LASSO uses a linear model L1 regularization (Tibshirani 1996), Zscore is a distance-based inference that relies on a Z-score approach (Seçilmiş *et al.* 2022a), LSCON utilizes normalized least square regression (Hillerton *et al.* 2022), and GENIE3 applies a tree-based ensemble method (Huynh-Thu *et al.* 2010). All these methods use the perturbation design matrix (so-called P-based approaches) during the inference as it was shown that such supervised methods outperform non P-based ones (Seçilmiş *et al.* 2022a). For each method, the inference was made on GRNs of two sparsities: 3 and 5 links per node on average (Martínez-Antonio *et al.* 2008, Marbach *et al.* 2012). Networks were created by using the scale-free network simulation tool included in GeneSPIDER with the datastruct.scalefree function where number of genes was set to 100 and sparsity was set to 3 or 5. For each sparsity two different signal-to-noise ratios (SNRs) were tested: 0.1 and 0.01, since real biological datasets are usually noisy (Eling, Morgan, and Marioni 2019) with SNR values that vary between 0.1 and 0.01 (Morgan *et al.* 2019, Seçilmiş *et al.* 2020). In this study, SNR is defined according to

(14) $$\mathrm{SNR}=\frac{\Sigma_{\min}(Y)}{\sqrt{\chi^{-2}(\alpha ,NM)\lambda }}$$

where $\Sigma_{\min}$ is the smallest singular value, Y the measured fold change in gene expression, $\chi^{-2}(\alpha ,NM)$ the quantile of the inverse $\chi^{2}$ distribution at level α with NM degrees of freedom (number of genes x number of experiments) and λ is the variance of the Gaussian noise (Hillerton *et al.* 2022).

From this definition of SNR, gene expression data $Y\in R^{N\times M}$ can be simulated using the equation

(15) $$Y=-A^{-1}P+E_{\mathrm{SNR}}$$

where $A\in R^{N\times N}$ is the interaction matrix representing the GRN, with eigenvalues with a negative real part, $P\in R^{N\times M}$ is the matrix of the perturbation design, i.e. defining which gene is perturbed in which experiment (0 or 1 values) and $E_{\mathrm{SNR}}\in R^{N\times M}$ is a matrix containing Gaussian noise with a standard deviation based on the chosen SNR.

**Figure 1.**
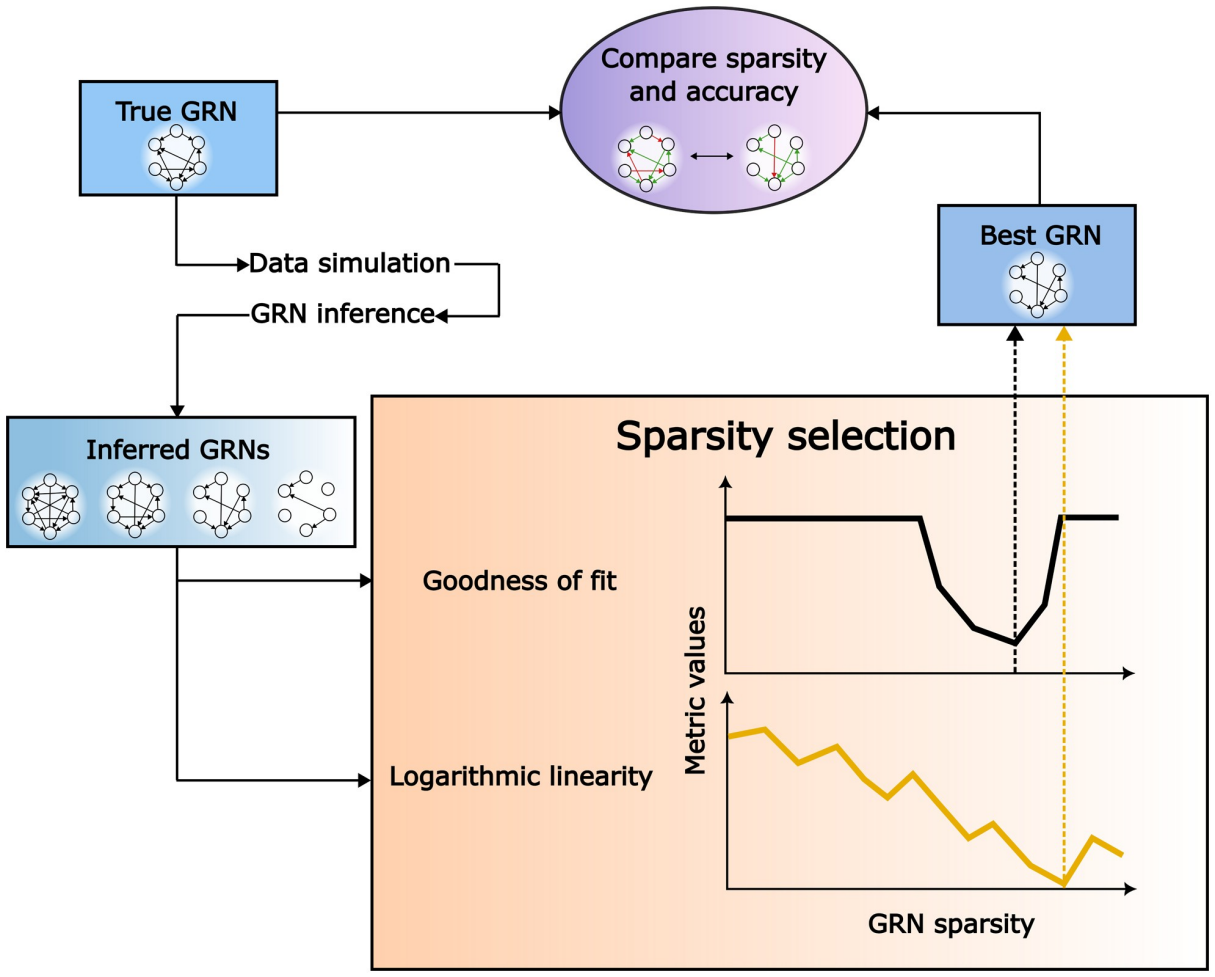
The general workflow of the analysis. Given a true GRN, data is simulated and GRNs are inferred. For all inferred GRNs the goodness of fit and logarithmic linearity metrics of power law adherence are calculated and used to predict the GRN with the optimal sparsity. These GRNs are then compared to the true GRN in terms of sparsity and accuracy.

For each combination of the GRN/data properties, three different GRNs were generated and tested. The GRNs contained 100 genes, and the data was generated using three replicates for each perturbed gene. The workflow is described in Fig. 1.

To measure accuracy the F-beta score (Goutte and Gaussier 2005) was used. This metric represents a weighted harmonic mean of precision and recall and is defined by the equation

(16) $$F_{\beta }=\frac{(1+\beta^{2})\cdot \text{precision}\cdot \text{recall}}{\beta^{2}\cdot \text{precision}+\text{recall}}$$

where

(17) $$\text{precision}=\frac{\text{true}\;\text{positives}}{\text{true}\;\text{positives}+\text{false}\;\text{positives}}$$

 

(18) $$\text{recall}=\frac{\text{true}\;\text{positives}}{\text{true}\;\text{positives}+\text{false}\;\text{negatives}}$$

A value of β>1 increases the influence of recall while the opposite increases the influence of precision. A value of β=2 was chosen to increase the weight of the recall because false positives far outnumber true positives in a sparse GRN.

## 3 Results

### 3.1 Evaluation on simulated data

To evaluate the proposed sparsity selection methods, we created benchmarking datasets with varying properties. For each sparsity, 3 and 5 links per node, three different GRNs containing 100 genes each were generated using GeneSPIDER, giving a total of six different GRNs. For each GRN, gene expression data was simulated for two different SNR levels, 0.1 and 0.01, using a perturbation design where each gene was knocked down in three replicates. Four different GRNI methods: LASSO, Zscore, LSCON, and GENIE3 were applied to each dataset, resulting in a total of 48 combinations of dataset and inference method. For each such combination the sparsity selection methods “goodness of fit” and “logarithmic linearity” were applied to evaluate the topology-based approach to optimal sparsity selection.

We first provide a detailed showcase presentation of the sparsity selection methods tested on GRNs inferred with LASSO and LSCON. The complete results on all methods and datasets are available in Supplementary Data, and are summarized later on. The methods were evaluated according to their ability to predict the best GRN in terms of sparsity and F2 score.

For visualization purposes, all goodness of fit metric values ≥104 were limited to this value, after which all values were min-max scaled, resulting in values between 0 and 1. As this is an optimization problem, the performance of the sparsity selection methods should be assessed based on the alignment between the sparsity at a metric minimum and either the true sparsity or the sparsity with the maximum F2 score.

**Figure 2.**
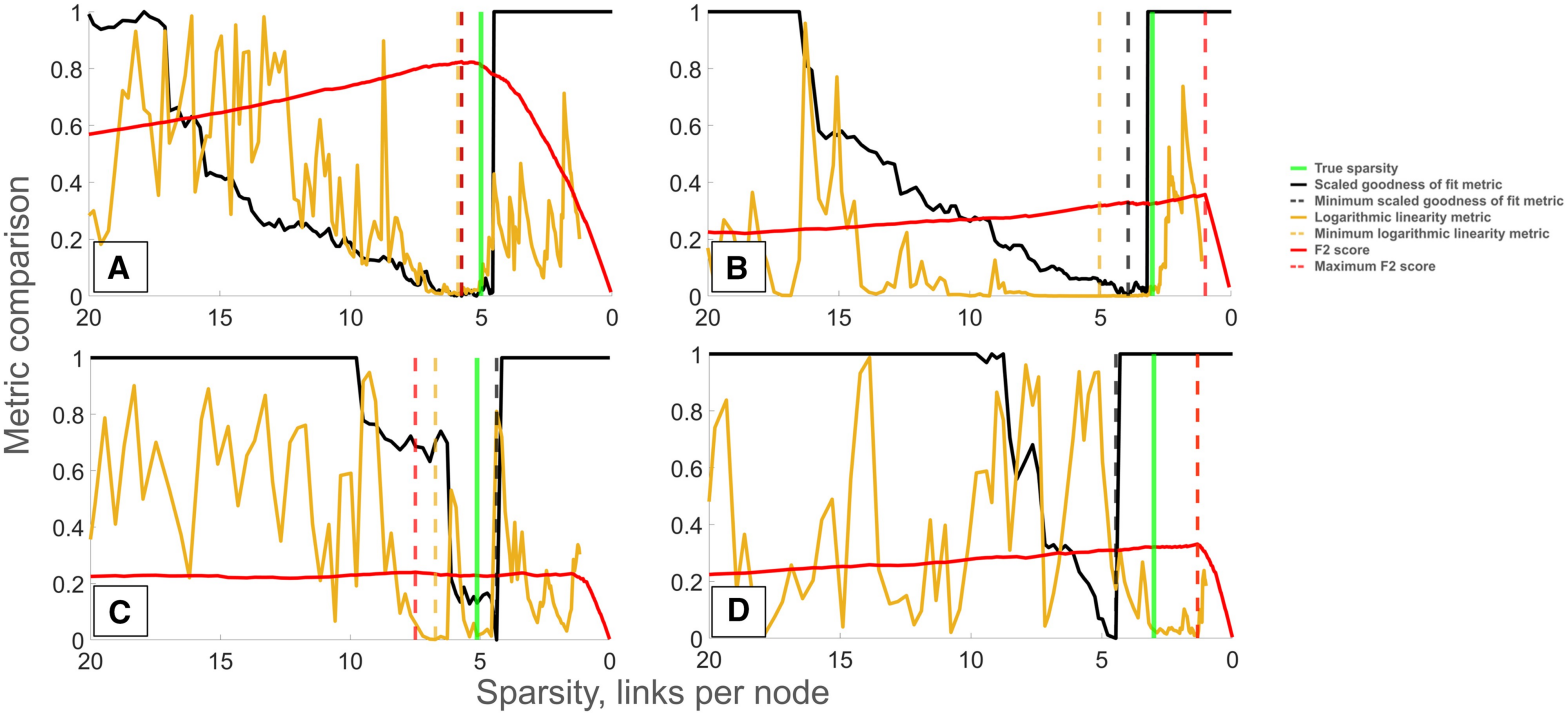
Comparison of sparsity selection methods applied to GRNs inferred with LASSO where the data was generated with SNRs 0.1 (A and B) and 0.01 (C and D) from GRNs with sparsities 3 (B and D) and 5 (A and C). The black line shows the rescaled goodness of fit metric, the light brown line shows the logarithmic linearity metric and the red line the F2 score. All metrics are shown as functions of the sparsity of the corresponding inferred GRN. The vertical dashed lines show minima of method metrics and maximum F2 score, respectively, and the vertical green line shows the true sparsity.

The results for LASSO are shown in Fig. 2. It can be seen that there sometimes is a considerable difference in sparsity between the inferred GRN that is best in terms of sparsity and the one with the highest F2 score. The goodness of fit metric behaves much smoother and with a better defined minimum, which leads to increased performance. In all cases it selected a GRN with a more correct sparsity, and in three out of four cases a higher F2 score than the logarithmic linearity method.

**Figure 3.**
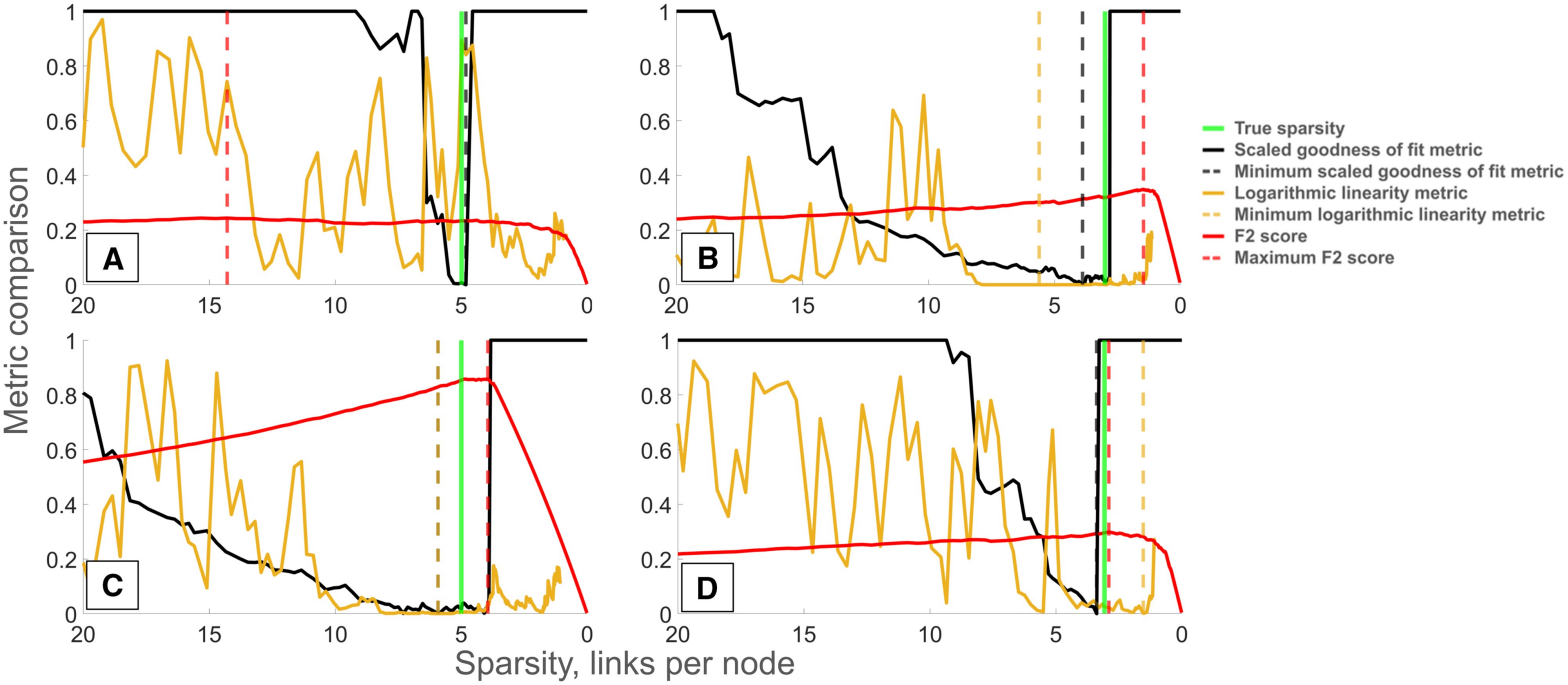
Comparison of sparsity selection methods applied to GRNs inferred with LSCON where data was generated with SNRs 0.1 (B and C) and 0.01 (A and D) from GRNs with sparsities 3 (B and D) and 5 (A and C). The black line shows the rescaled goodness of fit metric, the light brown line shows the logarithmic linearity metric and the red line the F2 score. All metrics are shown as functions of the sparsity of the corresponding inferred GRN. The vertical dashed lines show minima of method metrics and maximum F2 score, respectively, and the vertical green line shows the true sparsity.

The results from GRNs inferred with LSCON (Fig. 3) show the same pattern as the case with LASSO. However, the difference between the goodness of fit and logarithmic linearity methods in terms of sparsity prediction is slightly smaller.

**Figure 4.**
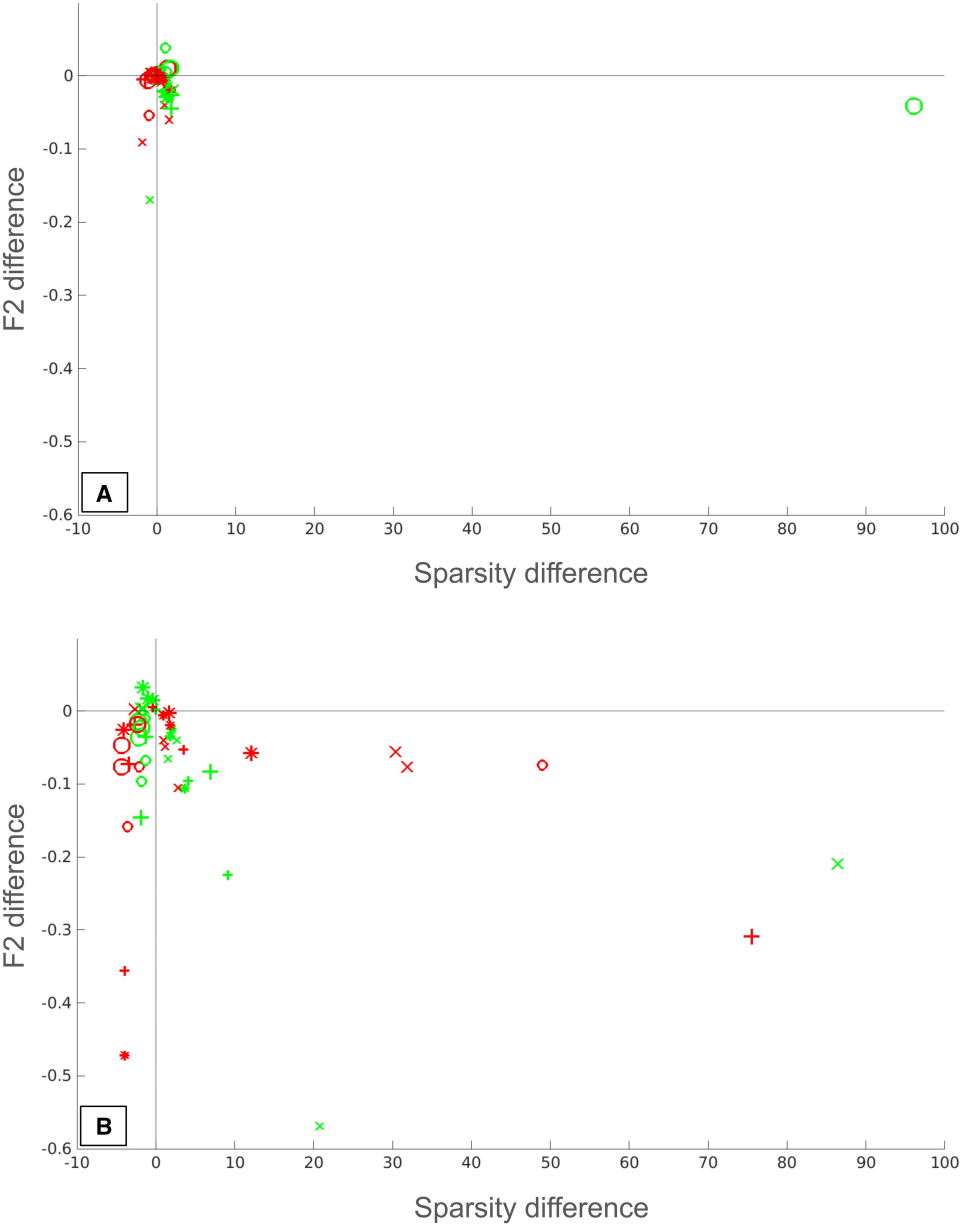
Comparison of prediction performance with respect to sparsity and accurateness score for the (A) goodness of fit metric and (B) logarithmic linearity metric. Data points are identified by shape (GRNI method), color (sparsity), and size (SNR). The vertical axis shows the difference in accurateness (F2 score), while the horizontal axis shows the difference in sparsity, between the selected GRN and the GRN with a sparsity closest to the true one.

To give an overview of the performance of both sparsity selection methods, we made summary scatter plots across all inference methods and datasets, see Fig. 4. Here each GRN selection is marked by a point showing the difference from the true sparsity versus the difference in accuracy relative to the inferred GRN closest in sparsity to the true GRN. We note that both metrics are well-balanced in terms of sparsity deviation, but that the goodness of fit metric is overall much closer to the optimum both in terms of sparsity and accuracy. A few outliers are present where the method selects a very dense GRN, indicating that in rare situations the metric can fail. This only happened once for the goodness of fit metric but 5–6 times for the logarithmic linearity metric. The sparsity of the true GRN has a strong influence on accuracy for the logarithmic linearity metric, in that the sparser true GRNs are predicted more correctly.

In order to investigate the performance of our algorithms with regards to GRNI method, we calculated the median absolute difference in sparsity between the predicted network and the inferred network with a sparsity closest to the true one, as shown by Table 1. We observe that LASSO has the best performance for both sparsity selection methods, while the relative performance of the other GRNI methods depends on the sparsity selection method. For example, GENIE3 has the poorest performance for goodness of fit while it is the second best method for logarithmic linearity.

**Table 1.** Summary comparison of sparsity selection performance for different GRNI methods.^a^

	LASSO	Zscore	LSCON	GENIE3
Goodness of fit	0.75	0.92	0.915	1.085
Logarithmic linearity	1.775	3.46	2.635	2.234

a To summarize all the results in Fig. 4, the median of absolute difference in sparsity between selected GRNs and the GRNs closest to the true sparsity is shown. GRNs were inferred by LASSO, Zscore, LSCON and GENIE3, and GRN selection was done with goodness of fit and logarithmic linearity.

**Figure 5.**
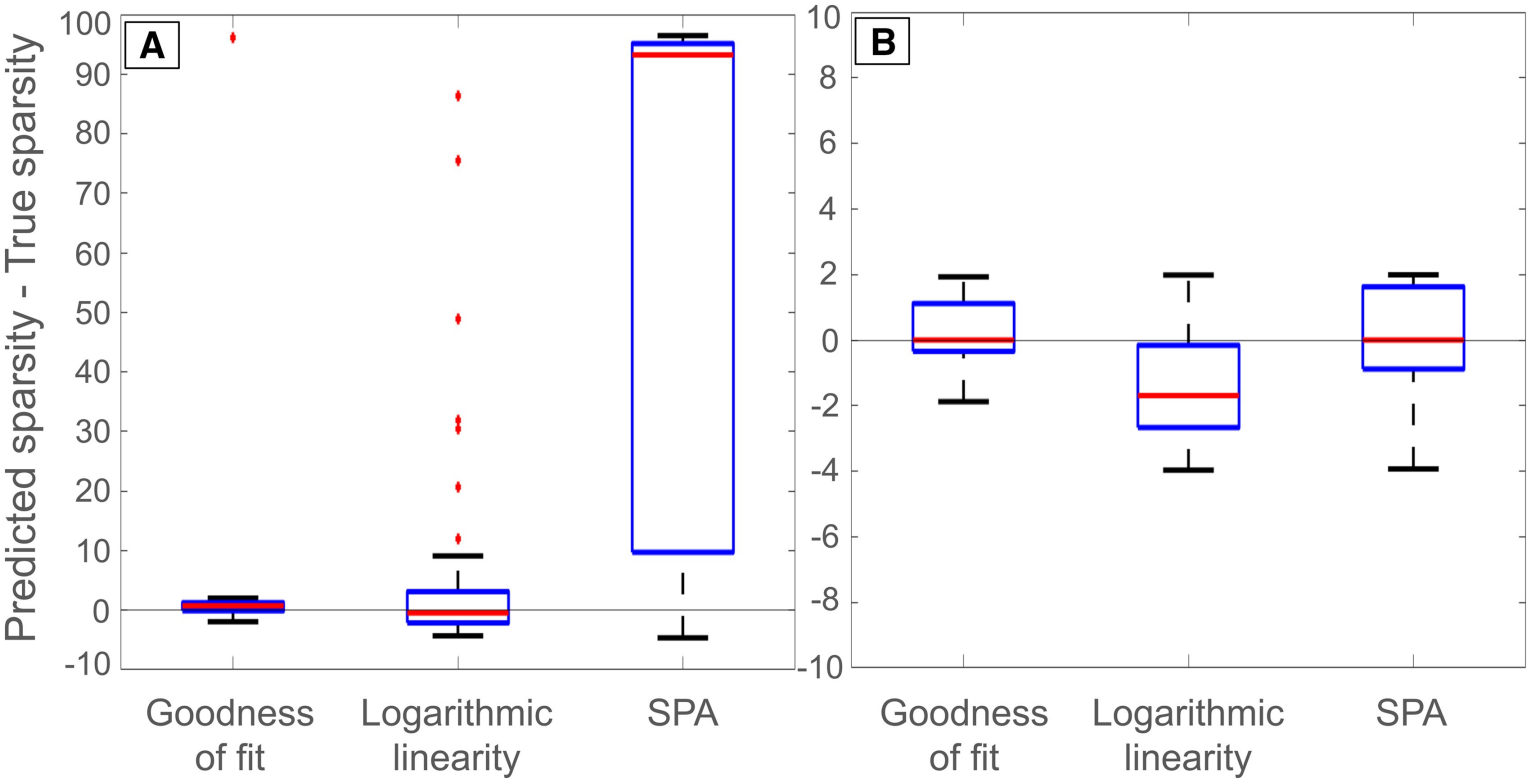
Comparison of the new methods to the SPA method. Boxplots showing distributions of sparsity prediction correctness for the goodness of fit, logarithmic linearity, and SPA methods on (A) all inferred GRNs and (B) inferred GRNs with sparsities between 1 and 5. GRN inference was done with LASSO, LSCON, Zscore, and GENIE3.

We further compared our two algorithms to the SPA method by measuring the difference between predicted and true sparsity. This was done on all GRNs, SNRs, and GRNI methods. By default, SPA only selects among inferred GRNs with a sparsity between 1 and 5, hence we performed the comparison both with this limitation and without it (Fig. 5). Without the limitation, SPA is dramatically outperformed by the new proposed metrics, but with the limitation the difference is relatively small. While all algorithms exhibit some errors in predictions, goodness of fit is the best among all methods, as its deviation from true sparsity is the smallest. We note that SPA has a strong tendency to overestimate sparsity.

### 3.2 Application to real datasets

#### 3.2.1 DREAM5 *E. coli* dataset

**Figure 6.**
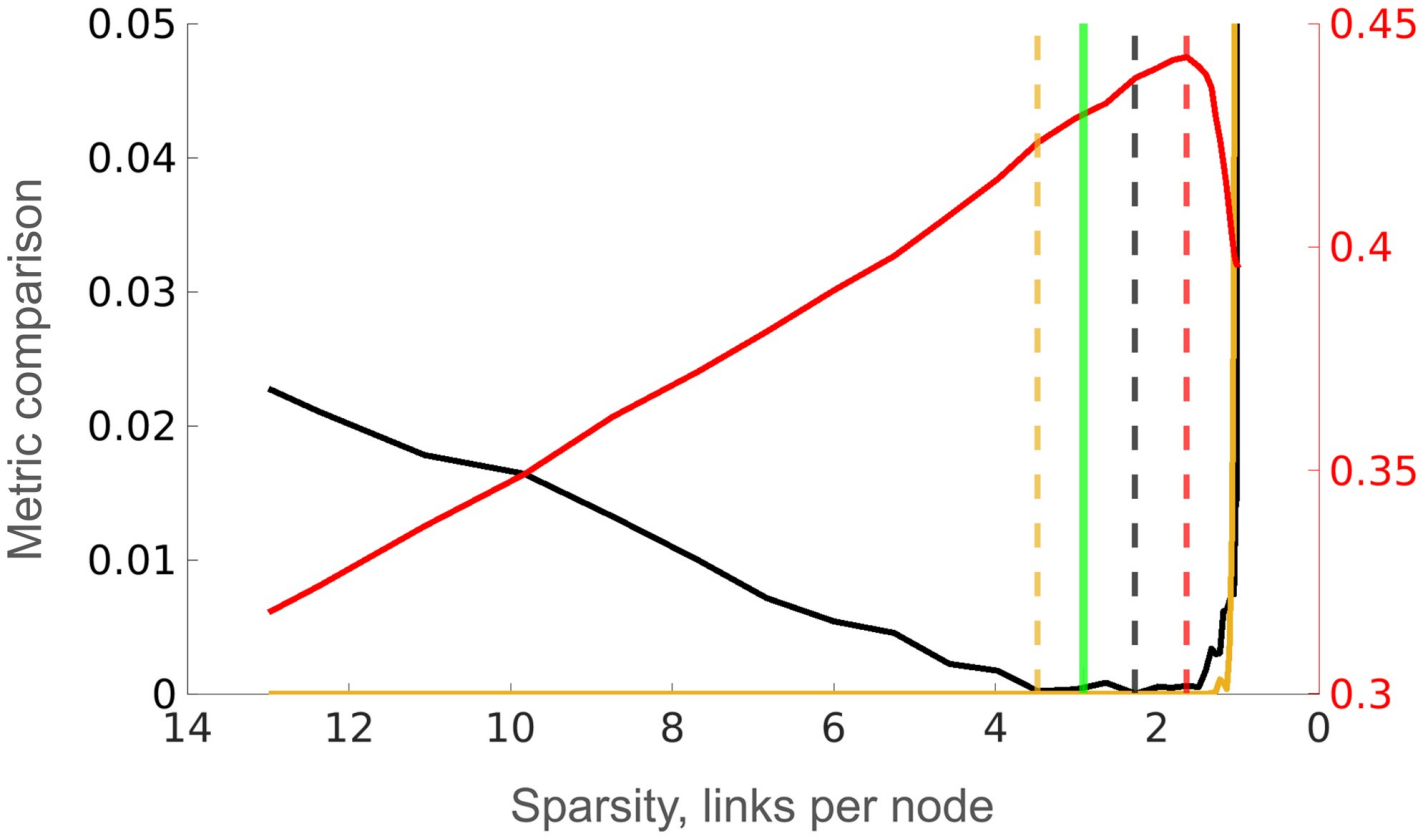
Application of the sparsity selection methods to GRNs inferred with GENIE3 on the DREAM5 challenge *E. coli* gene expression data. The black line shows the rescaled goodness of fit metric, the light brown line shows the logarithmic linearity metric and the red line the F2 score. All metrics are shown as functions of the sparsity of the corresponding inferred GRN. The vertical dashed lines show minima of method metrics and maximum F2 score respectively, and the vertical green line shows the true sparsity. The metrics (black and light brown lines) are scaled according to the left vertical axis and the F2 score (red line) is scaled according to the right vertical axis.

To test how the metrics perform on GRNs inferred from public real data, we applied them to the GRN inferred with GENIE3 on the DREAM5 challenge *E. coli* dataset (Marbach *et al.* 2012) containing gene expression data for 4511 genes. Since the gold standard GRN contains 1081 genes with nonzero in- or out-degrees, we only considered these genes. From the GENIE3 GRN we created 100 GRNs of different sparsity, ranging from the maximum to the minimum, by varying the threshold of minimum link confidence. The scale-free property of GRNs is linked to stability, and it has been shown that GRNs require a dominant negative diagonal (representing degradation or self-inhibition) to be stable (Zavlanos *et al.* 2011). Consequently, this was added to the inferred GRN and the gold standard. The sparsity selection methods were then applied using the same evaluation design as used on simulated data. As seen in Fig. 6, both methods predict GRNs with sparsities close to the true one. Similar to the results from simulated data, the GRN with the maximum F2 score is not optimal in terms of sparsity. However, the difference in F2 score between the two optimal GRNs is small.

#### 3.2.2 ENCODE datasets

**Figure 7.**
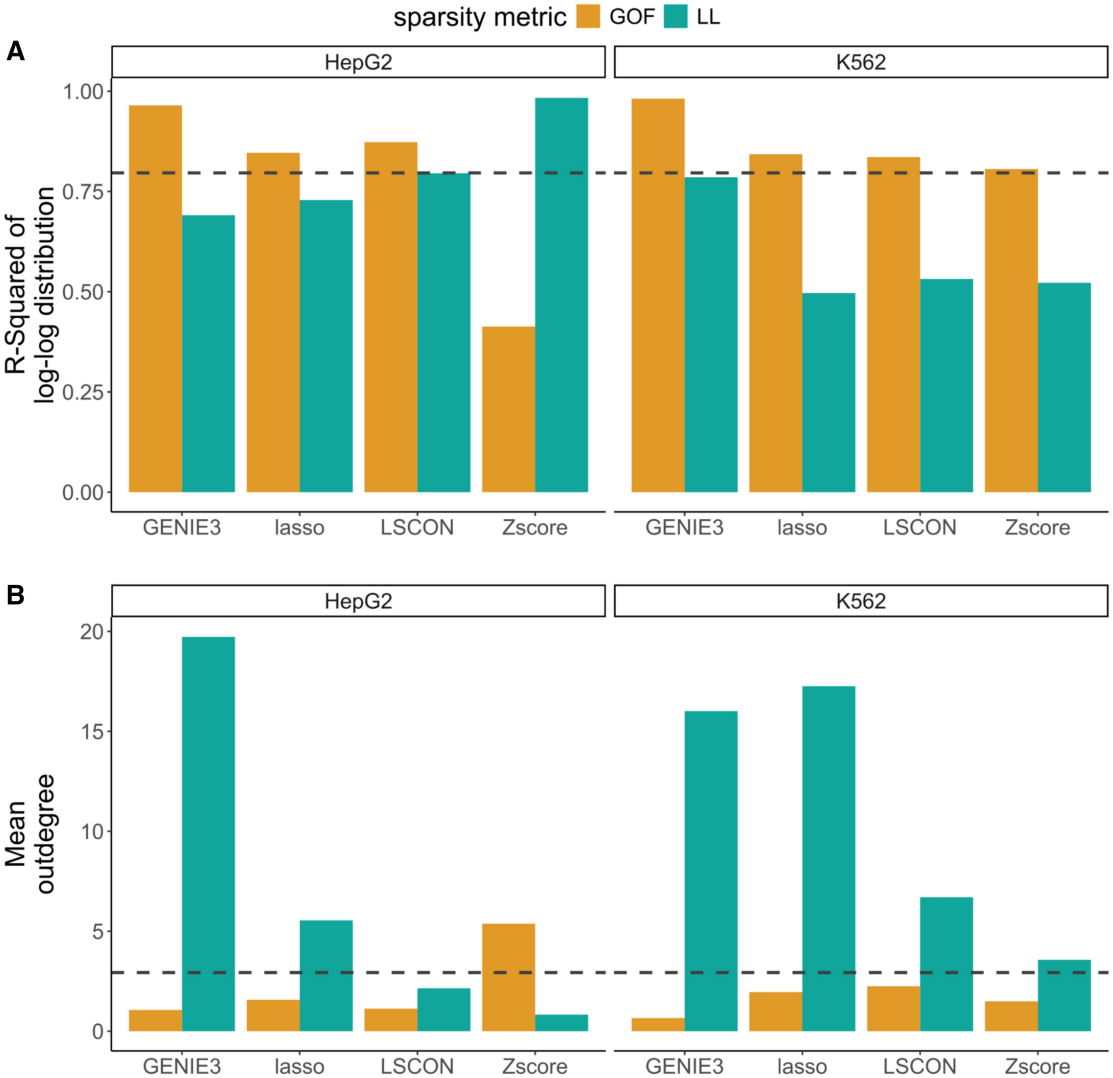
Evaluation of the two sparsity selection metrics goodness of fit (GOF) and logarithmic linearity (LL) applied to ENCODE expression data for cell lines HepG2 and K562. (A) Scale-freeness measured as the R-squared of the log-log distribution of out-degrees using linear regression (B) The average node out-degree. Dashed horizontal lines indicate a given property value for the TRRUST GRN.

To further test the metrics, we used publicly available ENCODE datasets with gene expression of the HepG2 and K562 cell lines (ENCODE Project Consortium 2012, Davis *et al.* 2018). As the real data lack a gold standard GRN, we here scored the GRN selected by the two metrics in terms of scale-freeness and average outdegree (Littman *et al.* 2023). This was done using the same GRNI methods as with the simulated data. For comparison, we marked the value of these properties in the human reference GRN from the TRRUST database (version 2) (Han *et al.* 2018). We observe that the selected GRNs have scale-free properties similar to TRRUST’s (Fig. 7A). This confirms the assumptions made for our metrics. However, logarithmic linearity tends to select GRNs with lower scale-freeness than goodness of fit. The mean outdegree for GRNs selected with the goodness of fit method is close to the TRRUST GRN (Fig. 7B), but logarithmic linearity tends to select denser GRNs. In the case of GRNs inferred with Zscore for HepG2, the goodness of fit selected the least scale-free GRN, while the logarithmic linearity selected a much more scale-free GRN. This is a good example of using these two approaches complementarily.

## 4 Discussion

The main result of this study is the development of two robust GRN selection metrics based on the scale-free topology hypothesis. We demonstrate that given a set of GRNs inferred at varying sparsities, the metrics can be used to accurately select a single GRN with a sparsity in the close vicinity of the true GRN that was used to generate the data for the inference. This is useful when real data is used for inferring a GRN, since a gold standard GRN is then normally lacking and the sparsity of a single best GRN needs to be estimated. Our method can thus support researchers in selecting a GRN near the biologically relevant sparsity, which is important for its biological properties and stability (Busiello *et al.* 2017).

As can be observed with both simulated and real data, by comparing the estimated sparsity metrics to the F2 score and true sparsity, the inferred GRN with the most correct sparsity does not in general obtain the highest F2 score. Using the F1 score the disparity was even larger. This effect may be caused by high noise that leads to false positive and negative links which can disrupt the alignment of accuracy and sparsity and make it impossible for a metric to predict the most correct GRN in terms of both the F2 score and sparsity. To some degree the sparsity selection method is only as effective as the applied GRNI method, meaning that the hypothesis of a scale-free topology is not as valid if the collection of inferred GRNs are poor. The ultimate goal of finding the true sparsity is only possible if the inference method is robust enough to ensure equivalence between maximum accuracy and true sparsity.

When comparing the performance at different noise levels, the goodness of fit metric in general isolates a much narrower set of plausible GRNs for high noise compared to low noise (Fig. 2), while such behavior cannot be seen for the logarithmic linearity metric. This could in part explain why the goodness of fit metric overall is the more reliable method.

A future direction might be to combine several metrics to obtain a more reliable consensus prediction, using e.g. a rule-based or neural network framework. Given the success of the presented methods, they can both serve as templates for future refinements.

## Data Availability

The supplementary figures are provided in our bitbucket repository (https://bitbucket.org/sonnhammergrni/powerlaw_sparsity/src/master/Graphs/).
